# In Situ Polyurea Integration for Self‐Healing, Durable Transparent Electromagnetic‐Interference Shielding Film

**DOI:** 10.1002/advs.75133

**Published:** 2026-04-02

**Authors:** Sinan Zheng, Shanyu Zhao, Gengjiang Yao, Yue Liu, Fei Pan, Mingrui Han, Zecheng Li, Shanbo Li, Jiurong Liu, Na Wu, Zhihui Zeng

**Affiliations:** ^1^ State Key Laboratory of Coatings for Advanced Equipment Key Laboratory for Liquid‐Solid Structural Evolution and Processing of Materials School of Materials Science and Engineering Shandong University Jinan P.R. China; ^2^ Laboratory for Building Energy Materials and Components Swiss Federal Laboratories for Materials Science and Technology, Empa Dübendorf Switzerland; ^3^ National Key Laboratory of Electromagnetic Effect and Security on Marine Equipment Wuhan P.R. China; ^4^ China Ship Development and Design Center Wuhan P.R. China; ^5^ Department of Chemistry University of Basel Basel Switzerland; ^6^ School of Chemistry and Chemical Engineering Shandong University Jinan P.R. China

**Keywords:** AgNWs/MXene, durability, polyurea, self‐healing, transparent EMI shielding film

## Abstract

Scalable fabrication of transparent electromagnetic‐interference (EMI) shielding films that combine durability with long‐range conductivity is in high demand for flexible electronics and the Internet of Things (IoT). Yet, scaling such shields remains challenging because of their vulnerability to chemical corrosion and mechanical deformation. Here, we report an in situ integration strategy by molecular engineering that retards polyurea curing kinetics to suppress crystallization, yielding a highly transparent, ultraflexible secondary‐amine polyurea (PuSA) with an ultra‐flat surface and self‐healing capability. The extended wetting‐to‐gelation window allows void‐free sandwiching of hierarchical conductive networks composed of 2D MXene nanosheets coated with 1D silver nanowires (AgNWs). The resulting PuSA@AgNWs/MXene@PuSA (AMP) film delivers commercial‐grade optoelectronics, rapid multi‐triggered self‐healing, and ultrabroadband EMI shielding from the gigahertz (GHz) to the terahertz (THz) range. Notably, it retains over 95% of its initial EMI shielding effectiveness (SE) under rigorous chemical, thermal, UV aging, and mechanical challenges. This work thus establishes a general, scalable route to translate fragile nanoconductors into high‐performance, rugged transparent shields with broadband protection and long‐term reliability in next‐generation displays and wearable electronics.

## Graphical abstract

A steric‐hindrance molecular engineering strategy uses dimethyl maleate (DMM) to convert poly(1,4‐butanediol) bis(4‐aminobenzoate) (PBDAB) into a precursor with reduced reactivity via Michael addition, thereby retarding the curing process to form a transparent, ultraflexible, and self‐healable secondary‑amine polyurea (PuSA) with a crosslinked network. The extended wetting‑to‑gelation window also enables the void‑free integration of silver nanowires (AgNWs) and MXene nanosheets, yielding a transparent, sandwich‐structured PuSA@AgNWs/MXene@PuSA (AMP) film. Combining impact/corrosion tolerance with ultrabroadband electromagnetic‐interference (EMI) shielding, this composite effectively addresses the long‐standing durability bottleneck of transparent EMI shielding films.

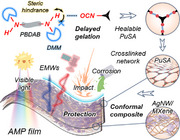

## 1 Introduction

The rapid evolution of next‐generation displays and electronics has intensified the demand for highly flexible, transparent conductive films (TCFs) capable of delivering robust electromagnetic interference (EMI) shielding [1, 2, 3]. Currently, various nanomaterials, including 1D metal nanowires [4] and carbon nanotubes (CNTs) [5], and 2D nanosheets such as graphene [6] and transition metal carbides/nitrides (MXenes) [7] with remarkable conductivity and large aspect ratio and specific surface area, have been increasingly explored with polymers to meet the simultaneous requirements of flexible optical transmittance and EMI shielding. However, as optical windows for human‐machine interaction, these TCFs must also be durable under harsh real‐world conditions over long service times, including chemical exposure, moisture/oxygen permeation, and mechanical abrasion and deformation, which is critical yet currently scarce [8, 9]. This scarcity stems from both the intrinsic vulnerability of the nanomaterials within the conductive layers and the lack of protective components that simultaneously provide effective mechanical support and chemical barrier properties for fragile percolative networks [10, 11, 12]. For instance, commonly employed polydimethylsiloxane (PDMS) is highly permeable to moisture [13], polyethylene terephthalate (PET) is prone to hydrolysis under humid conditions [14], and polymethyl methacrylate (PMMA) is limited by inherent brittleness [15]; even polyimides (PI), while chemically stable, often compromise optical clarity due to intrinsic coloration [16]. These limitations allow environmental permeants and mechanical deformation to induce oxidation, delamination, or structural fracture within the conductive network, resulting in pronounced increases in resistance and the eventual loss of EMI shielding effectiveness (SE) [17, 18]. More importantly, once the percolation pathway in TCFs is broken, it is difficult to re‐establish, causing irreversible electrical and EMI SE failure and an unnecessary waste of resources. Consequently, there is a pressing need for high‐performance yet durable transparent EMI shielding platforms that integrate mechanical toughness, environmental barrier capability, and intrinsic self‐healing potential, while remaining compatible with device‐level integration for real‐world service.

The inherent trade‐off between transparency and conductivity further amplifies the durability gap of nanostructured TCFs. To preserve optical transmittance, conductive layers in TCFs typically operate as sparse networks near the percolation threshold, rendering them sensitive to perturbations such as corrosion or microcracks [19, 20]. Moreover, although encapsulation or densification can delay degradation [21, 22], most reported TCFs remain fundamentally damage‐intolerant [23]. After scratching or bending‐induced cracking, the conductive pathways cannot be actively reconnected, leading to persistent increases in resistance and permanent SE decay [24, 25]. Furthermore, they are vulnerable to extreme mechanical impacts like ultrahigh‐frequency shockwaves, whose impulsive stresses readily trigger structural damage, thereby compromising the protection of underlying electronics [26]. Breaking this failure cascade requires a paradigm shift from single‐component optimization to a synergistic architectural design that combines a robust, stable conductive layer with a healable, high‐toughness flexible protective matrix. Polyurea emerges as an attractive candidate satisfying many of these criteria by leveraging its unique microphase‐separated structure [27, 28], wherein the rigid hard segments construct a dense barrier skeleton for promoting mechanical strength, the flexible soft segments enhance mechanical energy dissipation, and the polar urea linkages provide high‐density hydrogen bonding to strongly anchor the conductive network [29, 30]. Nevertheless, polyurea still struggles with limited flexibility and optical clarity [31], and its overquick polymerization kinetics can easily induce uncontrolled crystallization and domain mismatch, while rigid urea linkages restrict the molecular rearrangements essential for self‐healing [32, 33]. Therefore, a comprehensive strategy that combines molecularly engineered polyurea with an efficient conductive network design to decouple long‐term environmental durability from electrical and EMI shielding functionalities represents a promising yet challenging path toward truly rugged, self‐healable TCFs.

Here, we propose an in situ polyurea integration strategy to fabricate self‐healing, ultraflexible, and transparent EMI shields tailored for harsh environments. Using a modified, controlled‐gelling precursor of polyurea, we exploit an extended wetting‐to‐gelation window to form a highly flexible, ultra‐flat secondary‐amine polyurea (PuSA) with remarkable optical transparency. Furthermore, we construct a hierarchical conductive network composed of 2D MXene nanosheets coated with 1D silver nanowires (AgNWs) between PuSA layers, creating a sandwich‐structured PuSA@AgNWs/MXene@PuSA (AMP) film. The improved leveling and uniform microphase separation of PuSA reduce light scattering and suppress brittle domains. This results in enhanced flexibility and optical transmittance while maintaining polyurea's inherent strength. Consequently, the AMP film exhibits improved durability against mechanical deformation and chemical corrosion. Moreover, the robust interfacial adhesion firmly locks the fragile percolative network and suppresses crack growth, leading to high toughness and even remarkable shockwave resistance. In addition, the dense, reversible hydrogen‐bonding network enables bond reformation and chain diffusion across damaged interfaces, further supporting intrinsic self‐healing and service‐life extension. Notably, the AMP films deliver ultrabroadband EMI shielding across the measured gigahertz (GHz) and terahertz (THz) ranges while maintaining superior SE stability under diverse harsh conditions, significantly outperforming existing transparent EMI shields. Combined with the facile, scalable manufacturing of the AMP film, this work thus establishes a general strategy for rugged transparent EMI shielding films for next‐generation displays and wearable electronics.

## 2 Results and Discussion

**FIGURE 1:**
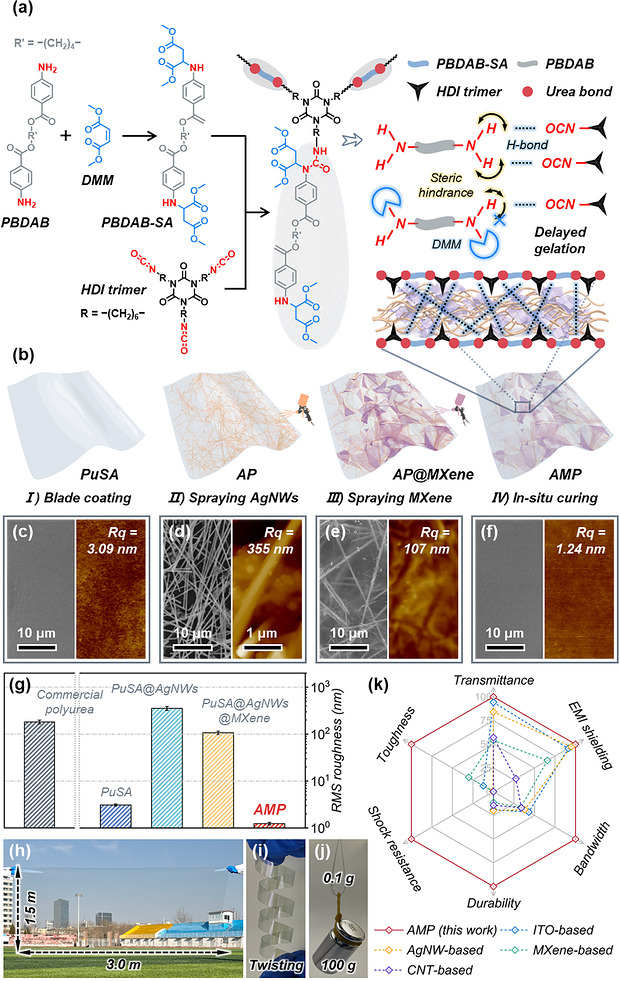
Molecular engineering and in situ integration of AMP films. (a) Synthetic route of PBDAB‐SA and PuSA. (b) Schematic of the in situ wetting to gelation process to assemble sandwich‐structured AMP films. (c–f) Representative AFM and SEM surface morphologies at different fabrication stages, showing PuSA‐enabled planarization of the conductive network. (g) Surface roughness statistics. (h) Photograph of a large‐scale AMP film. (i) Mechanical compliance demonstration. (j) Load‐bearing test (>1000× self‐weight). (k) Radar chart comparing the comprehensive performance of the AMP with previously reported transparent films.

To resolve the processing limitations of conventional polyurea and achieve an optically clear yet robust protective layer, we first molecularly engineered the amine‐terminated precursor poly(1,4‐butanediol) bis(4‐aminobenzoate) (PBDAB). As illustrated in Figure 1a, the primary amines of PBDAB were converted into secondary amines through a Michael addition with dimethyl maleate (DMM), yielding a secondary‐amine precursor (PBDAB‐SA). The conversion is verified by the characteristic red shift of the N–H stretching region (Figure S1) [34]. The steric hindrance and reduced nucleophilicity of PBDAB‐SA significantly retard the curing kinetics with HDI trimer (Figure S2), yielding PuSA. This kinetic retardation is pivotal as it shifts the morphology evolution from an uncontrolled, fast crystallization‐driven phase separation to a controlled, more homogeneous amorphous microphase separation. This transformation eliminates crystalline grain boundaries that strongly scatter light, suppresses brittle crystalline domains, and preserves broader, more dynamic interdomain interfaces for hydrogen‐bond rearrangement and chain diffusion, thereby jointly promoting transparency, toughness, and self‐healing [35, 36]. In addition, the slower gelation provides an extended window for wetting and leveling before solidification, which is essential for fully coating percolative nanoconductors without voids or surface defects. With this engineered PuSA, we fabricated the AMP films via an in situ wetting‐to‐gelation integration route (Figure 1b). Therein, a base PuSA layer was blade‐coated, followed by sequential spray deposition of 1D AgNWs and 2D MXene nanosheets, and then sealed by another in situ gelling PuSA top‐coat to form a compact sandwich structure. Cross‐sectional scanning electron microscopy (SEM) and element maps (C, Ti, and Ag) confirm that the hybrid conductive layer is tightly confined between two PuSA layers, with no interfacial gaps (Figure S3), indicating effective composition and strong interfacial adhesion. This design mechanically locks the percolative network, simultaneously provides a dense molecular barrier against moisture and oxygen, and relieves local stresses around the fragile nanoconductor junctions.

The microstructural advantages of in situ PuSA integration are directly reflected in the surface morphology and macroscopic mechanical properties. While the AgNW/MXene hierarchical network is successfully assembled (Figure S4), such sparse networks near the percolation threshold are intrinsically rough. Atomic force microscopy (AFM) and SEM comparisons at different fabrication stages (Figure 1c–f) show that the viscous PuSA precursor efficiently infiltrates the network, filling nanoscale valleys and conformally covering protruding nanowires and flakes, which markedly suppresses topographical fluctuations. As a result, the AMP film exhibits a substantially reduced nanoscale roughness (Figure 1g), outperforming both the uncoated network and films protected by unmodified pristine polyurea (Figure S5). The smoother surface reduces diffuse scattering at both the polymer‐air and polymer‐conductor interfaces, yielding high optical transparency and clearer visibility by suppressing diffuse scattering that would otherwise increase haze (Figure 1 h; Figure S6). Meanwhile, the amorphous microphase‐separated PuSA layer is highly compliant, allowing the AMP film to sustain repeated bending, folding, twisting, and rolling without cracking (Figure 1i; Figure S7). The sandwich‐structured film can also bear loads exceeding 1000 times its own weight (Figure 1j), demonstrating the mechanical robustness. The radar chart further highlights the balanced advantages of AMP across transparency, shielding performance, durability, and mechanical tolerance compared with previously reported ITO‐based and nanostructured TCFs involving AgNW‐, nanocarbon‐, and MXene‐based films (Figure 1k; Table S1).

**FIGURE 2:**
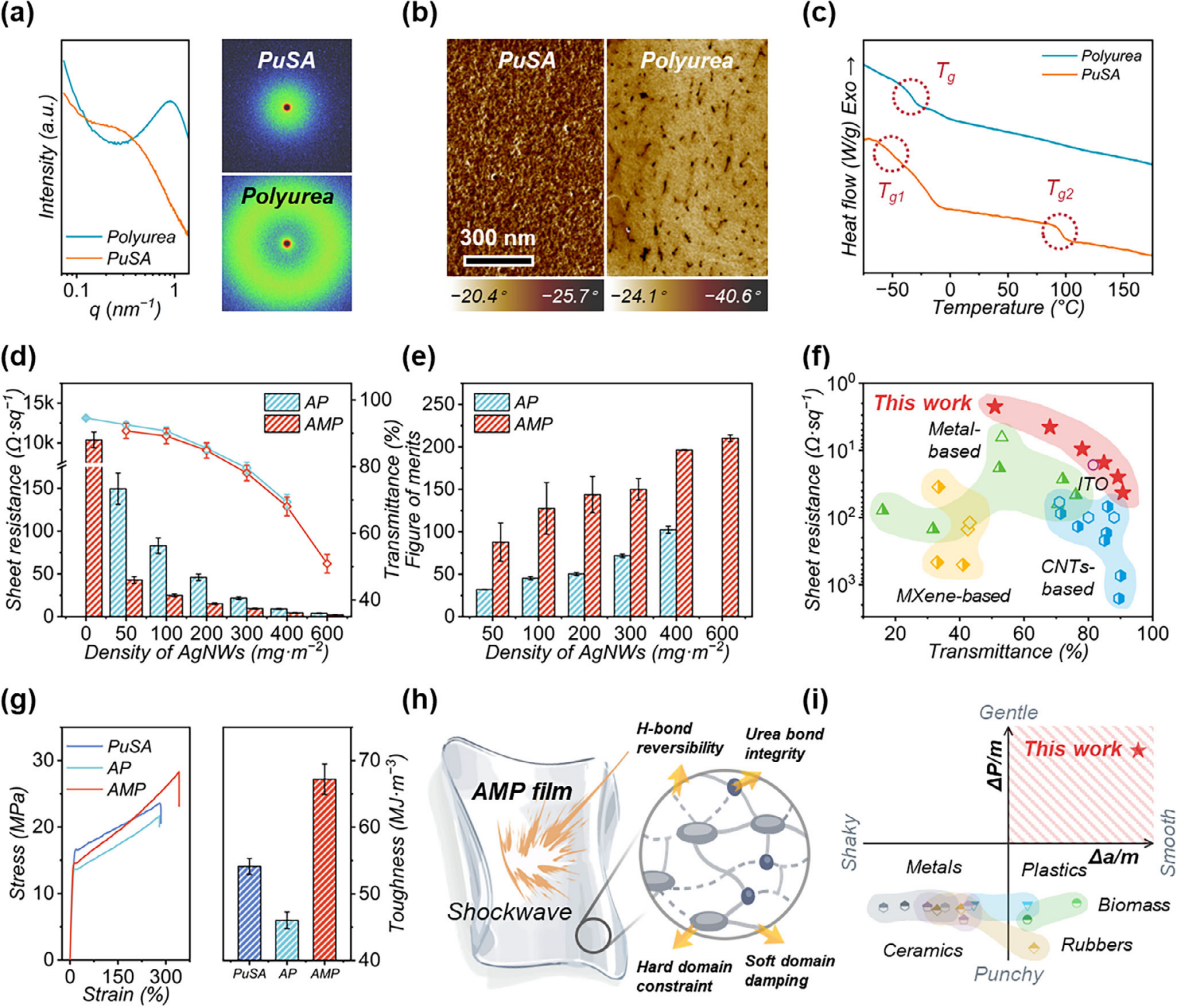
Optoelectronic origin and mechanical performance of AMP films. (a) 1D/2D SAXS profiles/patterns of pristine polyurea and PuSA. (b) AFM images illustrating the microphase morphology of pristine polyurea and PuSA. (c) DSC curves of pristine polyurea and PuSA. (d) Optoelectronic optimization enabled by MXene‐assisted junction bridging with minimal optical loss. (e) FoM comparison. (f) *Rs* vs. transmittance benchmark against previously reported TCFs and commercial ITO. (g) Tensile stress–strain curves and toughness comparison. (h) Schematic of the shockwave buffering mechanism. (i) Ashby plot of specific impact‐protection performance.

The high transparency of the AMP platform relies on both an optically clear protective matrix and a minimally scattering conductive layer. To elucidate the transparency advantage of PuSA over conventional polyurea, we examined its microphase separation characteristics. As shown in Figure 2a, pristine polyurea displays a pronounced correlation peak in the 1D profile and a distinct ring in the 2D pattern, indicating the presence of periodic nanostructures with strong electron density contrast. In contrast, after converting PBDAB to PBDAB‐SA, the PuSA sample exhibits much weaker and more diffuse scattering features. This suggests that the long‐range ordering is suppressed, and the hard domains become less crystalline and less periodic. AFM phase images (Figure 2b) confirm this trend in real space [37]. Pristine polyurea exhibits a sea‐island morphology with strong phase contrast, whereas PuSA displays a finer, more uniform texture with reduced contrast, indicative of smaller, dispersed domains with broader interfaces. The differential scanning calorimetry (DSC) curves (Figure 2c) further clarify the nature of these phases. In the measured temperature range, pristine polyurea exhibits only one glass transition (*T*_g_) associated with the soft segments, suggesting that the hard segments are locked in crystalline domains, where the dominant thermal event is melting at higher temperatures. Conversely, PuSA exhibits two distinct *T*_g_, indicating that both soft and hard segments remain amorphous and form separated microdomains [38]. This transition from crystalline to amorphous hard domains suppresses grain‐boundary scattering and minimizes refractive‐index mismatch, thereby explaining PuSA's high transparency alongside its mechanical robustness. On the conductive‐layer side, the hybrid AgNW/MXene design optimizes the transparency‐shielding balance by assigning complementary roles to the two components. The AgNW network provides the long‐range percolative backbone at low areal density and thus preserves optical transparency, whereas a small amount of MXene is introduced mainly to bridge AgNW junctions and enhance local electrical continuity. Since large MXene flakes can scatter light and reduce transmittance (Figure S8), excessive MXene is undesirable even if it slightly lowers sheet resistance (*Rs*). Therefore, we selected a minimum MXene density around the percolation knee point that preserves transmittance while effectively bridging junctions (Figure S9). In this regime, MXene nanosheets partially cover AgNW junctions, enlarging the effective contact area and providing additional current pathways. This bridging effect significantly reduces *Rs* with negligible impact on optical transmittance (Figure 2d) [39, 40]. Consistent with the visible‐light spectra of the AgNW@PuSA film (AP), the AgNW/MXene film on a PuSA substrate (AM), and the AMP film (Figure S10), the in situ PuSA infiltration also smooths the network and eliminates interfacial air voids, further minimizing optical loss. As a result, the optimized AMP film achieves a high figure of merit (FoM, Figure 2e) and a competitive Rs‐transmittance benchmark comparable to existing TCFs involving commercial ITO and previously reported metal‐, MXene‐, and nanocarbon‐based TCFs (Figure 2f; Table S2).

Beyond optoelectronic performance, transparent EMI shielding films frequently serve as interfaces for human‐machine interaction, where impact buffering and attenuation are critical for protecting underlying electronics. However, most TCFs that perform well under quasi‐static conditions remain susceptible to cracking under extreme shock conditions. Fortunately, the amorphous microphase‐separated PuSA layer endows the AMP films with outstanding mechanical robustness. The film exhibits high strength and ductility (Figure 2g), indicating that the embedded hierarchical network does not embrittle the polymer. This intrinsic toughness enables the film to withstand not only quasi‐static deformations but also impulsive events such as drops, collisions, and even shockwaves. The AMP architecture mitigates this failure mode by combining molecular‐level dissipation with structural confinement. The synergy between soft segments (acting as molecular dampers) and hard segments (providing mechanical constraints and physical crosslinks), tightly coupled through dense, reversible hydrogen bonds and strong urea linkages, forms a dynamic yet cohesive network that delays microcrack initiation and enables efficient energy dissipation (Figure 2h) [41, 42]. Moreover, the embedded AgNW/MXene network further assists shock buffering by introducing stiffness within the sandwich architecture, creating acoustic impedance mismatches that scatter the wavefront and attenuate the impulse energy. Consequently, the AMP film demonstrates excellent buffering capability against high‐frequency shockwaves (Figure S11). The Ashby plot (Figure 2i; Figure S12) highlights that AMP occupies a favorable region, combining strong impact protection with relatively low density, comprehensively verifying its mechanical reliability from tensile deformation to extreme shock mitigation.

**FIGURE 3:**
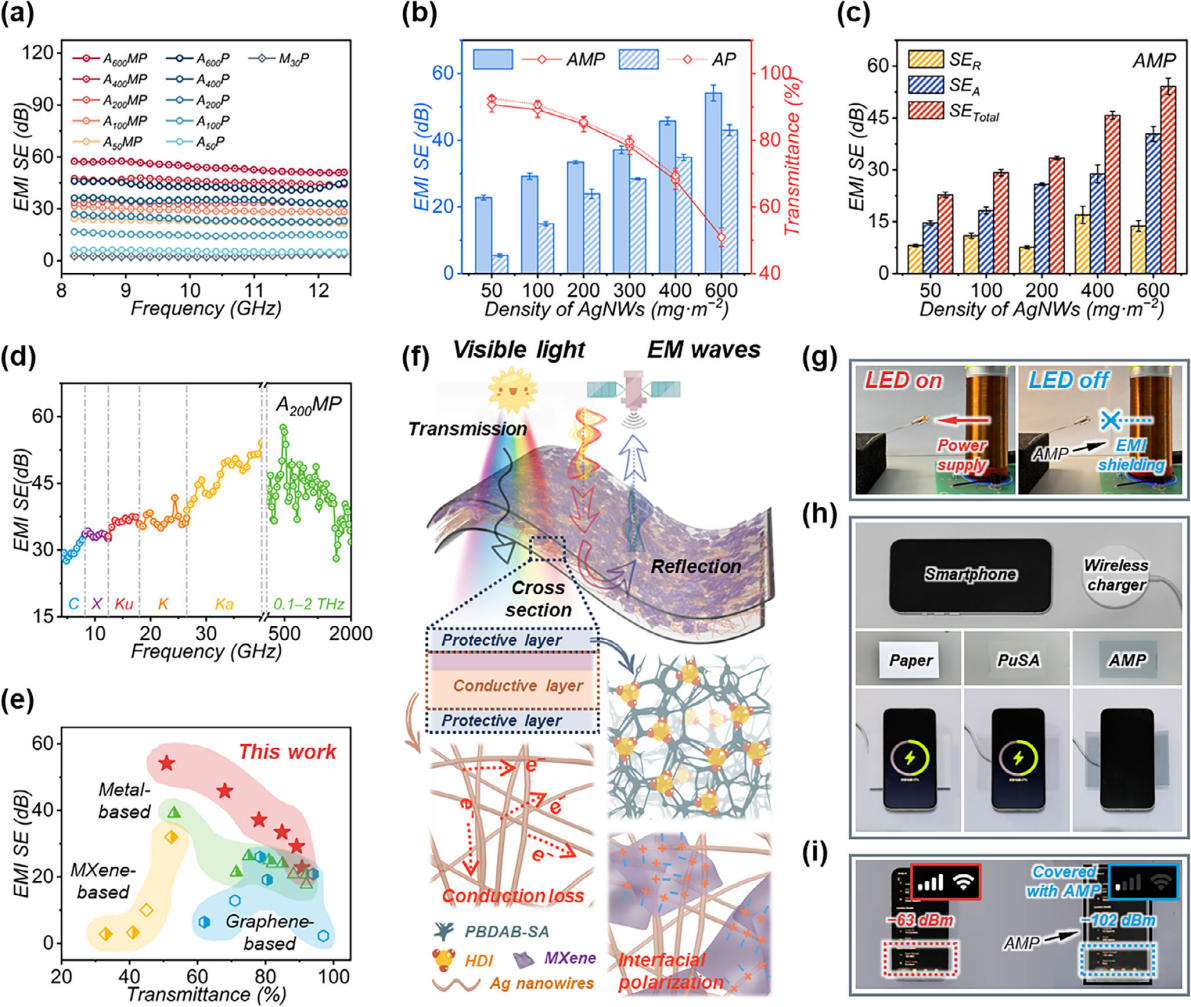
EMI shielding performance of AMP films. (a) X‐band EMI SE with different AgNW densities, highlighting hybrid‐network synergy. (b) Trade‐off between EMI SE and optical transmittance. (c) Deconvolution of total SE into SE_R_ and SE_A_ contributions. (d) Ultrabroadband EMI shielding in the measured 4–40 GHz and 0.1–2 THz ranges. (e) Benchmark of EMI SE and transparency vs. previously reported EMI shielding materials. (f) Schematic illustrating the transparent sandwich structure and EMI shielding mechanisms. Demonstration of (g) Tesla coil blocking, (h) wireless‐charging blocking, and (i) representative smartphone signal attenuation under the specific indoor ambient‐network conditions used in this work, highlighting the EMI shielding and attenuation capability of transparent AMP.

Building on its notable optoelectronic and mechanical foundation, the transparent AMP films deliver outstanding EMI shielding performance. In the X‐band (8.2–12.4 GHz), the EMI SE increases with AgNW density and exceeds the simple linear sum of the individual components (Figure 3a,b; Figure S13). At the microscale, MXene acts primarily as a conductive bridge that lowers contact resistance and improves electron transport within the sparse AgNW network, thereby strengthening the intrinsic shielding capability of the hybrid layer [43, 44]. At the macroscale, the sandwich structure mechanically stabilizes this percolative network, preserves electrical continuity, and prolongs the wave propagation path through internal reflection and multiple scattering, which together promote attenuation. The balance between EMI SE and optical transparency can thus be precisely controlled by adjusting the AgNW areal density (Figure 3b), offering a tunable route to meet specific requirements. Deconvolution of the total SE (SE_Total_) reveals that both reflection (SE_R_) and absorption (SE_A_) contributions increase with AgNW density (Figure 3c; Figure S14). The increased SE_R_ stems from the intensified impedance mismatch at the film‐air interface due to higher conductivity [45, 46], whereas the enhanced SE_A_ is associated with increased ohmic loss, interfacial polarization, and multiple scattering within the hierarchical network [47, 48, 49]. Despite the higher SE_A_, the impedance mismatching derived from the high conductivity of AMP films leads to the dominant reflection coefficient (Figure S15), which is consistent with the characteristics of highly conductive EMI shields. Notably, this high EMI shielding performance is achieved in both measured spectral windows, with SE values exceeding 30 dB from 4 to 40 GHz and surpassing 50 dB in the 0.1–2 THz range at a thickness of merely 100 µm (Figure 3d; Figure S16). Benchmarking against state‐of‐the‐art transparent EMI shields confirms that AMP offers a superior combination of high transparency, shielding efficiency, and broadband operation (Figure 3e; Figure S17, Tables S3 and S4). Figure 3f illustrates the underlying mechanism, highlighting how the optically transparent sandwich architecture of AMP stabilizes the conductive network while maximizing electromagnetic (EM) wave attenuation via coupled reflection and absorption.

These shielding characteristics translate into practical protection for electronics operating in diverse electromagnetic environments. Specifically, the AMP film effectively blocks electromagnetic coupling in radiative fields between a Tesla coil and an LED, as evidenced by the LED extinguishing when shielded (Figure 3g). Regarding near‐field coupling, the film's continuous percolation pathways and high conductivity effectively suppress magnetic induction, thereby preventing wireless charging when inserted between a charger and a smartphone (Figure 3h). Finally, the film significantly attenuates cellular and Wi‐Fi signals in the communication frequency domain when a mobile phone is enclosed within it (Figure 3i). Under the specific indoor test conditions used here, the received signal intensity decreased by 39 dB (from −63 to −102 dBm), corresponding to a nearly 8000‐fold reduction in received power, which underscores the film's superior shielding capability. Since this demonstration depends on the phone model, operating band, and local electromagnetic environment, we present it as representative application‐level evidence rather than a universal attenuation value. Together, these demonstrations corroborate the frequency‐domain metrics with practical protection tests. In practical service scenarios, the main advantage of AMP is that it combines optical transparency with durable, recoverable EMI shielding in a single platform, rather than offering only a high peak SE under ideal conditions.

**FIGURE 4:**
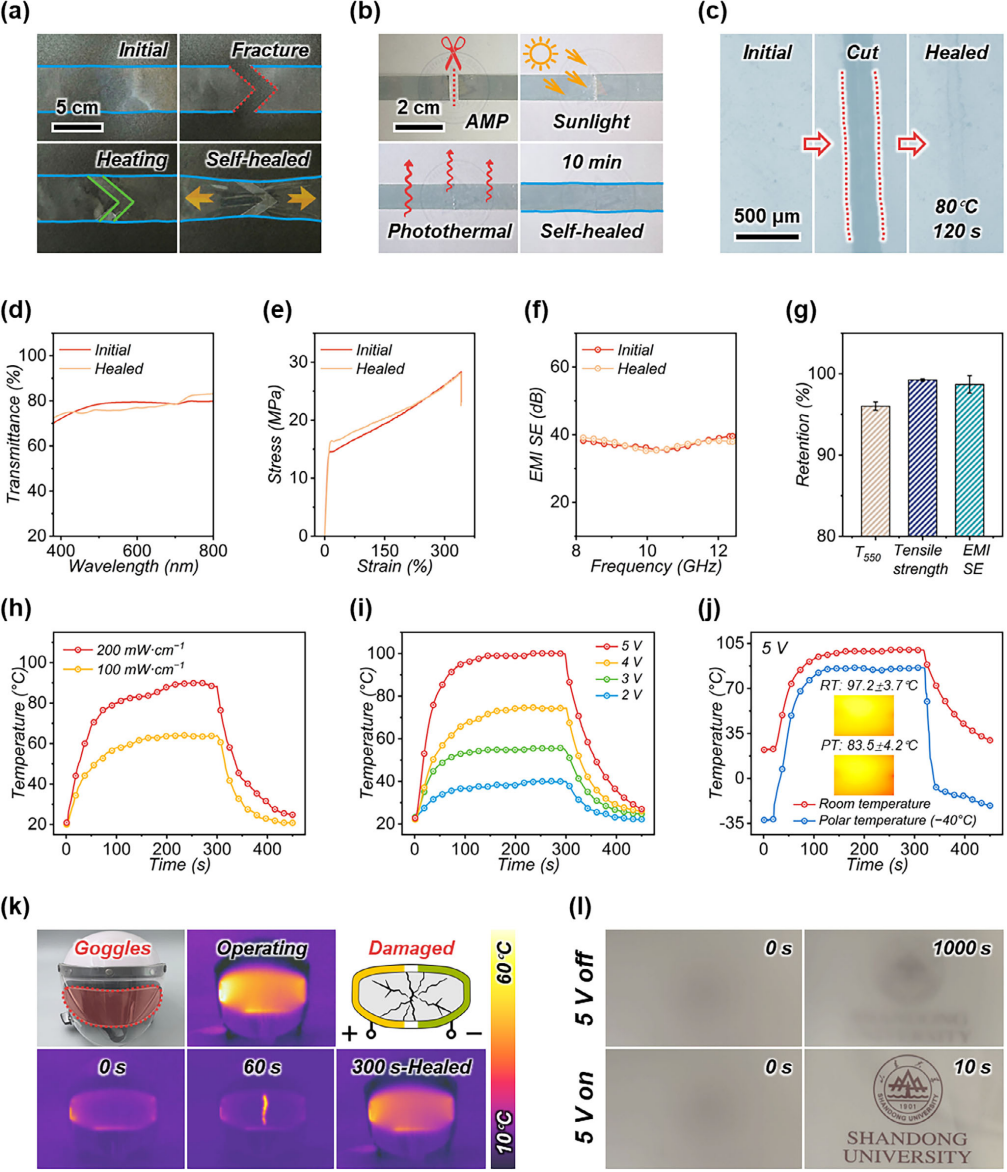
Mechanistically coupled self‐healing and multifunctional heating of AMP films. Photographs showcasing (a) thermal self‐healing process and (b) light‐triggered self‐healing process. (c) Optical microscopy showing the recovery of integrity after the self‐healing process. Recovery of (d) transparency, (e) mechanical strength, (f) EMI SE, and (g) their retention. (h) Photothermal and (i) Joule‐heating response under applied voltages. (j) Joule heating performance under normal and cryogenic conditions. The demonstration showcases the AMP films for (k) helmet‐visor integration and recovery of heating after damage, and (l) rapid defogging with visibility restoration.

The irreversible loss of conductive pathways following mechanical damage, such as scratches or cracks, is a key failure mode in percolative transparent conductors. The AMP film addresses this critical issue through its dynamic reversible hydrogen‐bonding network, which endows it with intrinsic self‐healing capabilities. The healing process, driven by bond exchange and chain interdiffusion across damaged interfaces, can be triggered by mild thermal activation via external heating (Figure 4a), electrical Joule heating, or photothermal heating from the hybrid conductive layer itself (Figure 4b), allowing remote and spatially selective healing. In this sense, the conductive network is mechanistically coupled to self‐healing because its Joule and photothermal heating directly provides the localized thermal trigger for recovery. After healing, the film regains its structural integrity (Figure 4c) and restores optical transparency, mechanical strength, and EMI shielding performance to levels comparable to those of the pristine state (Figure 4d–g). This self‐healing capability is critical for maintaining environmental durability, particularly in outdoor and wearable applications where complex stress conditions typically accelerate microcrack growth, opening diffusion pathways for oxygen and moisture. While the compact crosslinked network acts as an intrinsic barrier against oxidants, the self‐healing mechanism actively contributes to long‐term stability by autonomously re‐sealing surface micro‐defects. This prevents them from evolving into long‐lived permeation channels during aging.

The rationally designed MXene/AgNW hybrid network endows the AMP films with remarkable photothermal and electrothermal response capabilities. This dual functionality not only enables efficient thermal‐ and light‐triggered self‐healing, but also allows the films to serve as transparent photo‐ and electrothermal heaters. Under 1 sun illumination, the film rapidly reaches a steady‐state temperature exceeding 60°C (Figure 4h), providing a stimulus that can also assist photothermal healing when needed. Under electrical bias, AMP shows fast Joule heating and reaches a saturation temperature of 100°C within 150 s at 5 V (Figure 4i). The linear I‐V behavior confirms stable ohmic contact within the hybrid network (Figure S18) [50], and the steady‐state temperature scales linearly with the square of the applied voltage (V^2^), enabling predictable temperature control (Figure S19). The heating output can be adjusted by the AgNW density (Figure S20) and remains stable during long‐term operation (Figure S21). Notably, AMP retains electrothermal functionality even under cryogenic conditions (Figure 4j), highlighting its robustness in extreme environments. These combined features form a functional loop demonstrated in a helmet visor application. The AMP‐integrated visor warms rapidly to prevent fogging while retaining high transparency. Crucially, if the visor is scratched, the heating function can trigger self‐healing (Figure 4k), ensuring rapid defogging and restoring clear visibility even after damage (Figure 4l).

**FIGURE 5:**
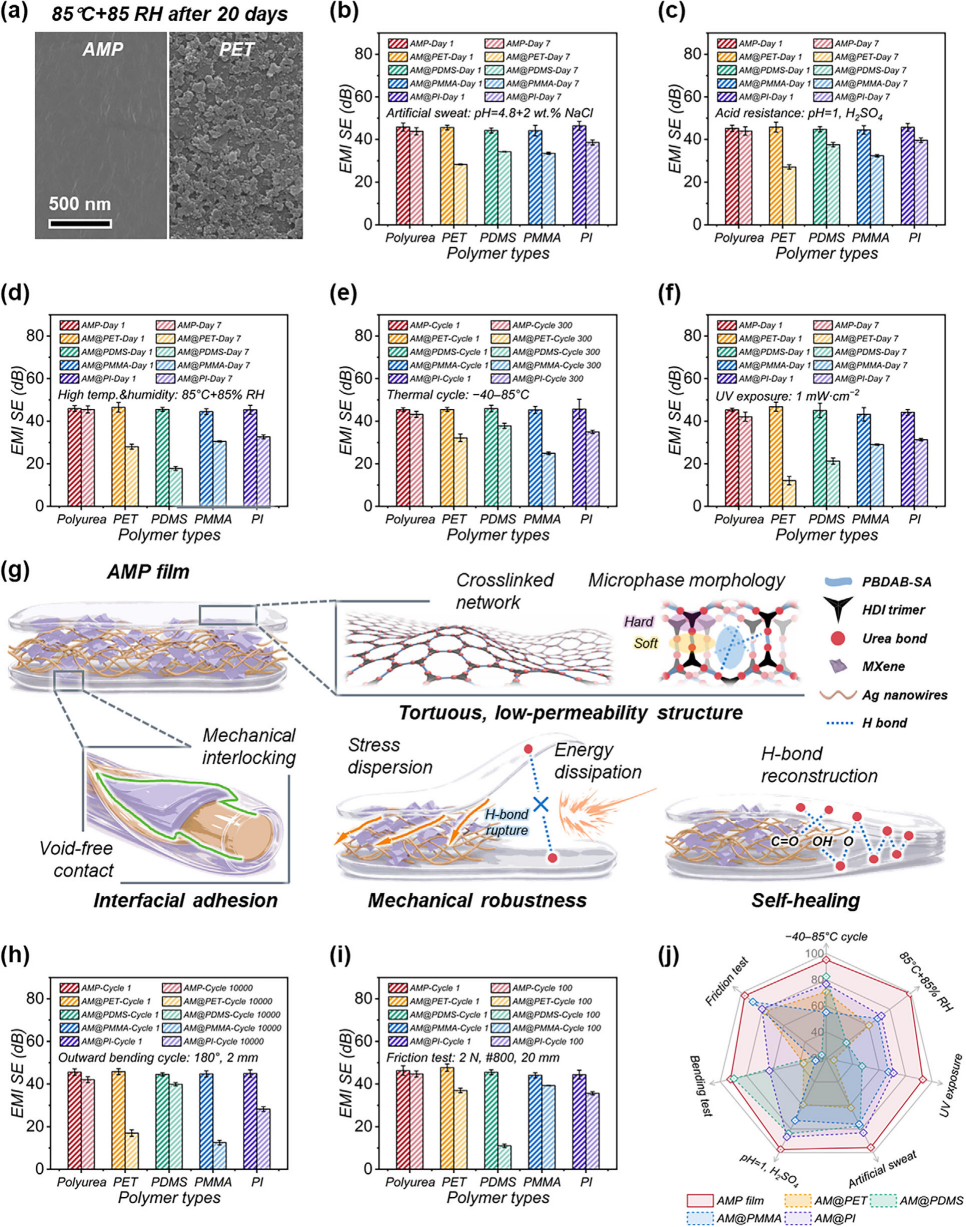
Durability of AMP films under harsh conditions and proposed mechanisms. (a) SEM morphologies of AMP and PET‑protected films after harsh aging at 85°C and 85% RH for 20 days, demonstrating corrosion protection superiority. (b–f) Retained EMI SE after representative chemical, thermal, and UV challenges for AMP and other polymer‐based TCF systems. (g) Schematic illustration distinguishing the roles of void‐free encapsulation, interfacial adhesion, sandwich confinement, and self‐healing in long‐term protection. (h,i) Retained EMI SE after representative mechanical challenges. (j) Radar plot summarizing EMI SE retention for AMP vs. counterpart films protected by other typical polymers.

Finally, we evaluated the long‐term environmental durability of the AMP film, addressing a key bottleneck for nanostructured transparent EMI shields. The kinetics‐driven in situ wetting‐to‐gelation process enables the precursor to infiltrate fully and conformally composite with the porous network, establishing a void‑free, low‑permeability barrier with tortuous diffusion pathways to retard the ingress of corrosive species. Strong interfacial adhesion between PuSA and the AgNW/MXene network then suppresses local debonding and preserves electrical contact during environmental attack and deformation. In addition, the sandwich confinement mechanically locks the percolative skeleton, limiting crack opening, out‐of‐plane distortion, and network rearrangement under repeated loading. Accordingly, after aging at 85°C/85% RH for 20 days, the AMP film remains intact, whereas the PET‐protected film shows severe oxidation and corrosion (Figure 5a). Beyond this accelerated damp‐heat test, we conducted comprehensive quantitative evaluations to challenge the film's limits. As shown in Figure 5b–f and Figures S22–S26, the AMP film retains over 95% of its initial SE under artificial sweat, acid exposure, thermal cycling, and UV irradiation. Under mechanical challenges, the H‑bond‑rich PuSA matrix disperses and dissipates stress and provides damage tolerance through dynamic network reconstruction (Figure 5g), enabling high SE retention during repeated bending and abrasion (Figure 5 h,i; Figures S27 and S28). Meanwhile, intrinsic self‐healing helps reseal microdefects before they develop into persistent permeation channels. In sharp contrast, films protected by other commonly employed polymers such as PET, PDMS, PMMA, and PI undergo catastrophic degradation under comparable tests (Figure 5j). This exceptional stability collectively validates the AMP strategy, demonstrating that the integrated PuSA matrix effectively resolves the intrinsic instability of nanostructured TCFs, ensuring reliable long‐term operation in harsh environments.

## 3 Conclusion

In summary, we have successfully addressed the longstanding conflict between high optoelectrical performance and environmental reliability in TCFs via a kinetics‐driven in situ integration strategy. By molecularly engineering the precursor to retard reaction kinetics and suppress crystallization, we convert the brittle, hazy polyurea into a durable, flexible, optically clear film with rapid self‐healing capacity. Moreover, this controlled wetting‐to‐gelation processing enables defect‐free assembly of the AgNWs/MXene hybrid network, enhancing its durability against permeants and stress concentrations while maintaining low‐resistance pathways through MXene‐assisted bridging. As a result, the AMP film with high optoelectrical properties not only demonstrates reliable transparent heating and broadband EMI shielding but, more importantly, exhibits post‐damage recovery of structural integrity and electrical continuity, unlike traditional TCFs. By retaining over 95% performance under thermal, chemical, and mechanical stress, this work validates the effectiveness of molecular‐level protection. Beyond the specific AgNW/MXene system, this kinetics‐controlled, ambient‐process offers a general, scalable strategy for transforming fragile nanoconductors into durable, transparent shields for flexible displays, thereby bridging the gap between high‐performance optoelectronics and long‐term reliability in practical applications.

## Conflicts of Interest

The author declares no conflicts of interest.

## Supporting information




**Supporting File**: advs75133‐sup‐0001‐SuppMat.docx.

## Data Availability

The data that support the findings of this study are available from the corresponding author upon reasonable request.
