# Supplementary material for: In Situ Polyurea Integration for Self‐Healing, Durable Transparent Electromagnetic‐Interference Shielding Film
Source: Adv Sci (Weinh). 2026 Apr 2;13(36):e75133. doi: 10.1002/advs.75133 (PMC13317791; doi:10.1002/advs.75133)
Supplement: Supplementary file 1 — Supporting File: advs75133‐sup‐0001‐SuppMat.docx. [file ADVS-13-e75133-s001.docx]

Supporting information

**In-Situ Polyurea Integration for Self-Healing, Durable Transparent Electromagnetic-Interference Shielding Film**

Sinan Zheng^1^, Shanyu Zhao^2, *^, Gengjiang Yao^3, 4^, Yue Liu^1^, Fei Pan^5^, Mingrui Han^1^, Zecheng Li^3, 4^, Shanbo Li^3, 4^, Jiurong Liu^1, *^, Na Wu^6, *^, Zhihui Zeng^1, *^

^1^ State Key Laboratory of Coatings for Advanced Equipment, Key Laboratory for Liquid-Solid Structural Evolution and Processing of Materials, School of Materials Science and Engineering, Shandong University, Jinan 250061, P.R. China

^2^ Laboratory for Building Energy Materials and Components, Swiss Federal Laboratories for Materials Science and Technology, Empa, Dübendorf, Switzerland

^3^ National Key Laboratory of Electromagnetic Effect and Security on Marine Equipment, Wuhan 430064, P.R. China

^4^ China Ship Development and Design Center, Wuhan 430064, P.R. China

^5^ Department of Chemistry, University of Basel, Basel CH-4058, Switzerland

^6^ School of Chemistry and Chemical Engineering, Shandong University, Jinan 250100, P.R. China

^*^ Corresponding author

E-mail: zhihui.zeng@sdu.edu.cn (Z.H. Zeng); na.wu@sdu.edu.cn (N. Wu); jrliu@sdu.edu.cn (J.R. Liu); shanyu.zhao@empa.ch (S.Y. Zhao)

**Experimental Section**

1. Materials

The following reagents were used in this study: silver nitrate (AgNO_3_), polyvinylpyrrolidone (PVP; Mw 55,000 and 360,000), ethylene glycol (EG), and ferric chloride (FeCl_3_) for the synthesis of silver nanowires (AgNWs). Ti_3_AlC_2_ MAX phase powder (400 mesh), lithium fluoride (LiF), and hydrochloric acid (HCl) for Ti_3_C_2_T_X_ MXene synthesis. Poly(1,4-butanediol) bis(4-aminobenzoate) (PBDAB) and dimethyl maleate (DMM) were used to prepare the secondary-amine precursor (PBDAB-SA). PBDAB-SA and hexamethylene diisocyanate (HDI) trimer were used to form the polyurea and polyurea-SA (PuSA) matrices. All chemicals were of analytical grade and used as received without further purification.

1. Synthesis of Silver Nanowires

Silver nanowires with high aspect ratios were synthesized via a modified polyol reduction method. In a typical synthesis, a mixture of PVP (0.05 g of K30 and 0.10 g of K90) was dissolved in 22 mL of ethylene glycol (EG) within a three-neck flask. After adding a catalytic amount of FeCl_3_ solution (2.5 mL, 0.6 M in EG), the mixture was heated to a stable temperature of 140℃. An EG solution containing AgNO_3_ (3 mL, 60 mg·mL^−1^) was then introduced dropwise to initiate nanowire growth. The reaction was allowed to proceed for 60 minutes after the solution turned gray, during which stirring was halted. The reaction was quenched with ethanol, and the resulting AgNWs were purified through repeated cycles of centrifugation (5000 rpm, 10 min) and washing with deionized water. The final product was redispersed in an aqueous solution at 1.5 mg·mL^−1^ for subsequent use.

1. Synthesis of Large-Flake Ti_3_C_2_T_X_ MXene Nanosheets

High-quality, large-flake Ti_3_C_2_T_X_ MXene was prepared from a Ti_3_AlC_2_ MAX phase precursor by adapting an optimized mild etching method. In a typical synthesis, 0.75 g of the Ti_3_AlC_2_ particles were etched using a minimally intensive layer delamination (MILD) approach. The etching was performed in a solution of 2.4 g of LiF dissolved in 30 mL of 9 M HCl at 50℃ for 48 hours. The resulting multilayered MXene was washed with deionized water via centrifugation (1500 rpm, 5 min) until the supernatant reached a neutral pH (~6). The washed sediment was then delaminated into single- or few-layered nanosheets through gentle agitation (manual shaking and vortexing) in deionized water, yielding a stable, dark-green aqueous dispersion of large-flake MXene.

1. Fabrication of AgNW/MXene@PuSA (AMP) Films via a Controlled In-Situ Wetting-to-Gelation Process

The AMP transparent conductive films were fabricated via a kinetics-controlled in-situ wetting-to-gelation integration strategy enabled by molecular engineering of polyurea:

Step 1: Base Layer Preparation and Conductive Network Deposition. A base PuSA layer was first coated and cured to serve as a flexible transparent substrate. The synthesized AgNW dispersion and the MXene ink were then sequentially spray-coated onto the PuSA substrate, forming a rough but highly conductive 1D/2D hybrid network.

Step 2: Precursor Infiltration and Planarization. The HDI trimer and PBDAB-SA (prepared by Michael addition of PBDAB with DMM) were thoroughly mixed to form the PuSA precursor. This liquid precursor was then immediately cast onto the rough, as-sprayed conductive network. Benefiting from the retarded curing kinetics, the precursor maintained low viscosity and excellent fluidity within the wetting-to-gelation window, enabling both conformal infiltration/encapsulation of the porous network and efficient planarization of the film surface.

Step 3: In-Situ Curing. The entire assembly was allowed to cure at ambient temperature. This solvent-free, in-situ curing process solidified the top PuSA encapsulation layer, locking in an optically smooth surface and creating a robust, seamlessly integrated sandwich structure with the conductive network fully embedded.

Step 4: Final Film. The resulting fully encapsulated AMP film features an ultra-smooth surface, exceptional environmental stability, and strong interfacial adhesion between the conductive layer and the PuSA matrix.

For clarity, the film notations used in this Supporting Information are defined as follows: PuSA (polyurea-SA matrix), AP (AgNW@PuSA film, AgNW network without MXene), AM (AgNW/MXene film on a PuSA substrate without the top encapsulation layer), AMP (fully encapsulated AgNW/MXene@PuSA sandwich film).

1. Characterization

The morphological and structural properties of the materials and films were investigated using scanning electron microscopy (SEM; Zeiss MERLIN Compact), transmission electron microscopy (TEM; JEOL 2200 FS), and atomic force microscopy (AFM; Bruker ICON3). Optical and electrical characteristics were determined by UV-vis spectrophotometry (PerkinElmer Lambda 1050+) and a four-point probe system (RST-8), respectively. The electromagnetic interference (EMI) shielding effectiveness in the GHz range was measured using a vector network analyzer (Agilent N5244A) over the X-band and Ka-band frequency ranges. Terahertz (THz) shielding performance was evaluated by terahertz time-domain spectroscopy (THz-TDS) in the 0.1–2 THz range. Unless otherwise specified, the film thickness used for EMI shielding measurements in both the GHz and THz ranges was 100 μm. Joule heating performance was evaluated by applying a DC voltage with a DC power supply (UNI-T UTP1306S), while the surface temperature was monitored with an infrared thermal imaging camera (FLIR E60).


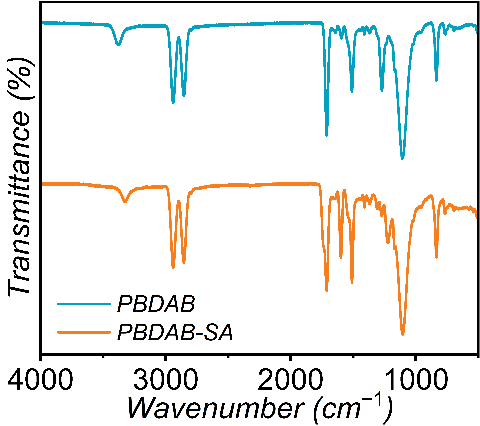


**Figure S1.** FTIR spectra of the pristine PBDAB precursor and the modified PBDAB-SA precursor.


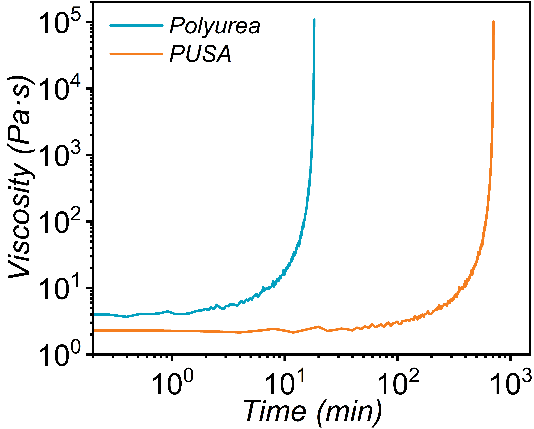


**Figure S2.** Time-dependent viscosity evolution during curing for pristine polyurea and polyurea-SA (PuSA), highlighting the retarded curing kinetics of PuSA.


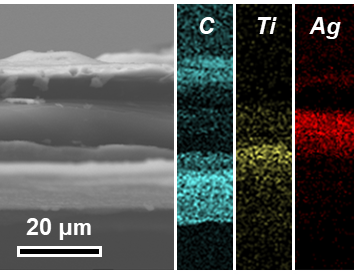


**Figure S3.** Cross-sectional SEM image and corresponding EDS elemental mappings (C, Ti, Ag) of the AMP film.


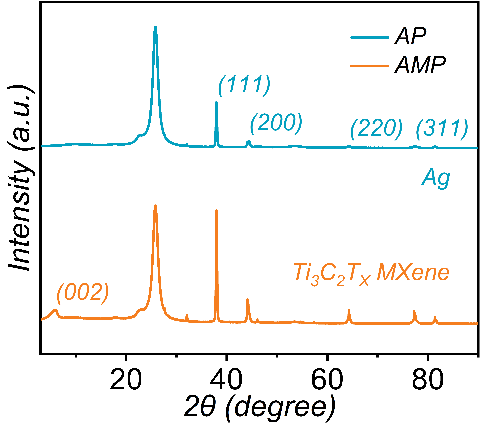


**Figure S4.** XRD patterns of AP and AMP films, verifying the crystal structures of Ag and Ti_3_C_2_T_X_ MXene components.


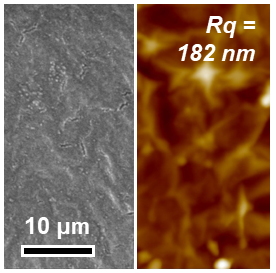


**Figure S5.** SEM and AFM height images of the bare AgNW/MXene network covered by conventional polyurea.


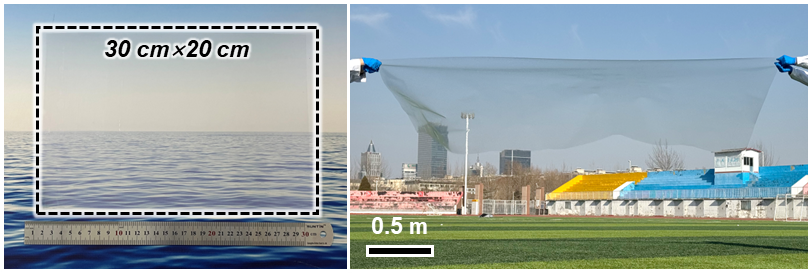


**Figure S6.** Digital photographs of the AMP film demonstrating its high optical transparency and excellent mechanical flexibility.


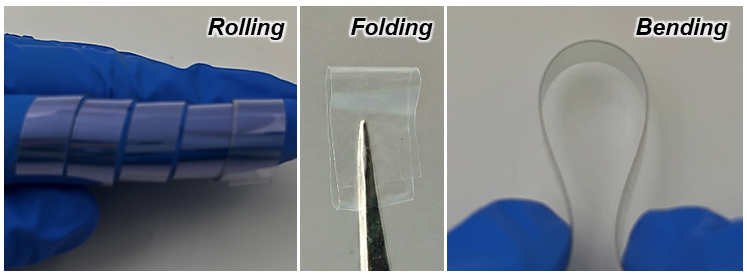


**Figure S7.** Mechanical compliance demonstration, including rolling and bending.


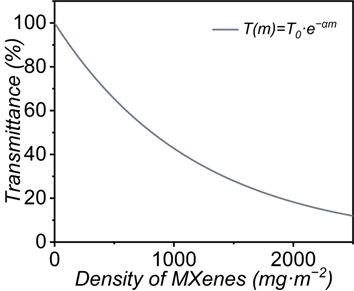


**Figure S8.** Optical transmittance of composite films as a function of MXene deposition density.


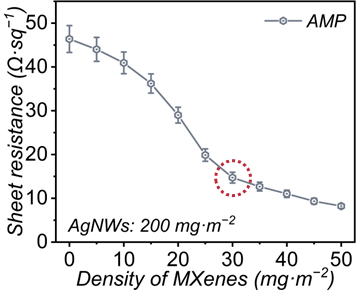


**Figure S9.** Sheet resistance of AgNW/MXene hybrid networks as a function of MXene deposition density (AgNWs fixed at 200 mg·m^−2^). The circled region indicates the optimized MXene loading used in this work.


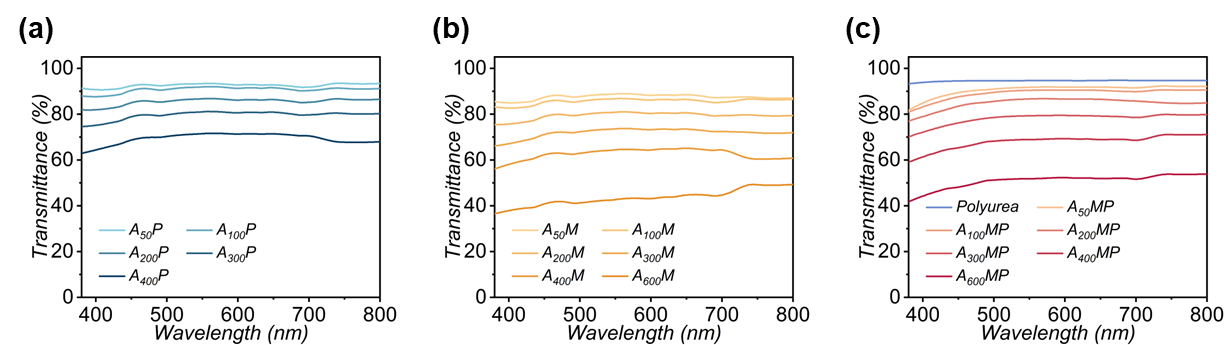


**Figure S10.** Visible-light transmittance spectra of (a) AP films, (b) AM films, and (c) fully encapsulated AMP films.


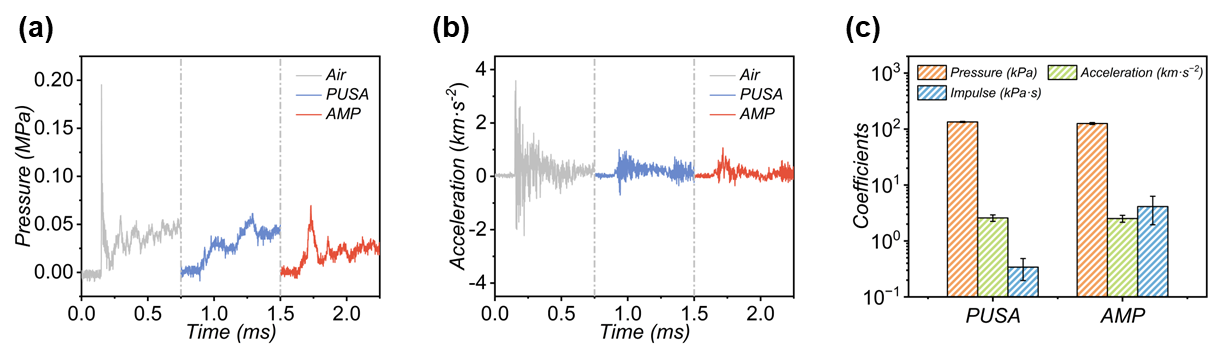


**Figure S11.** Shockwave impact attenuation performance. (a) Pressure-time, (b) acceleration-time, and (c) comparison coefficients for air, PuSA, and the AMP film, demonstrating the superior shockwave buffering capability enabled by the tough, energy-dissipating PuSA matrix.


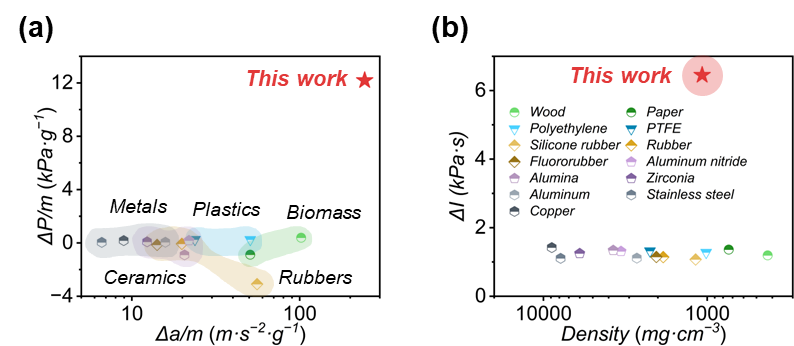


**Figure S12.** Ashby plot comparison of specific impact protection performance for different protective materials.


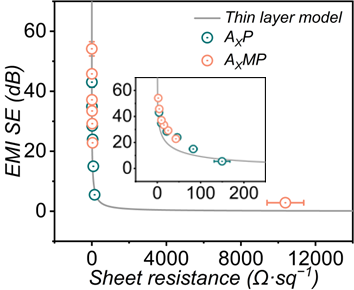


**Figure S13.** X-band EMI SE of AP and AMP films with varying AgNW densities, compared with the theoretical thin-layer model.


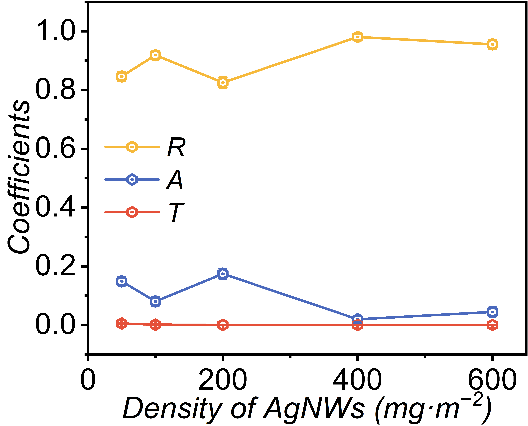


**Figure S14.** Electromagnetic power coefficients (R, A, T) of AMP films as a function of AgNW density.


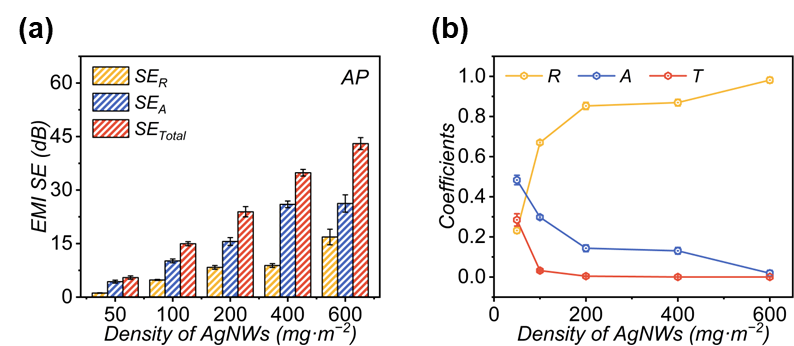


**Figure S15.** (a) Frequency-dependent total shielding effectiveness (SE_Total_), reflection loss (SE_R_), and absorption loss (SE_A_) of AP films in the X-band. (b) Electromagnetic power coefficients (R, A, T) of AMP films as a function of AgNW density.


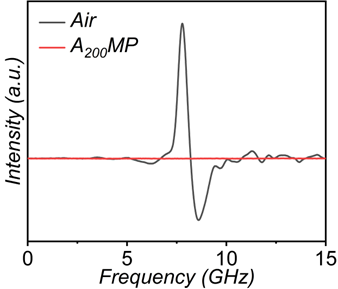


**Figure S16.** Time-domain THz signal comparison between air (reference) and the AMP film in the 0.1–2 THz range.


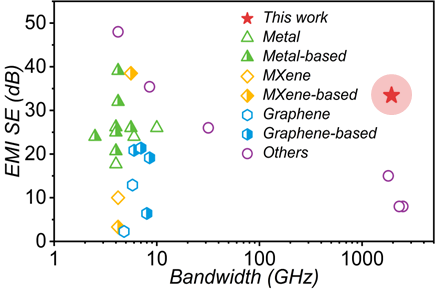


**Figure S17.** Comparison of EMI shielding effectiveness versus operating bandwidth for reported transparent shielding materials.


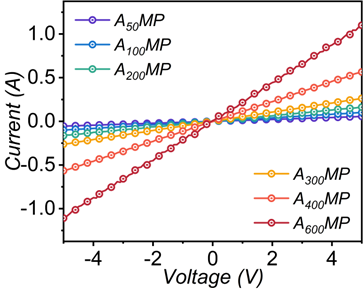


**Figure S18.** Current-voltage characteristics of the AMP transparent heater.


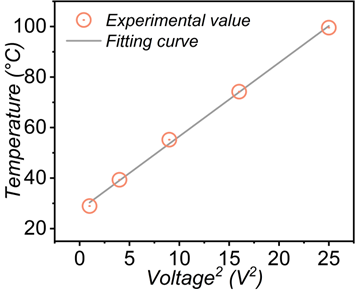


**Figure S19.** Saturation temperature of the AMP heater as a function of the square of applied voltage.


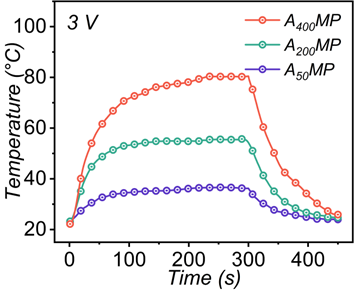


**Figure S20.** Saturation temperatures of AMP heaters with different AgNW areal densities under identical driving voltages.


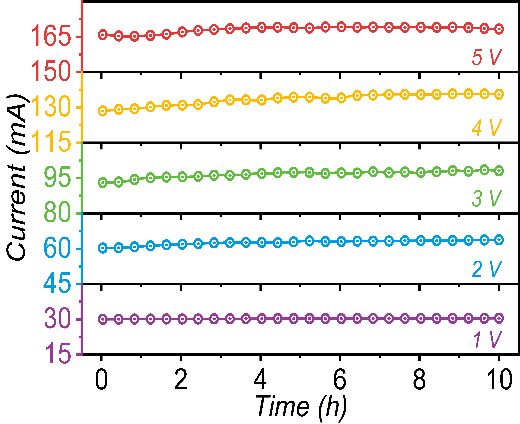


**Figure S21.** Long-term electrical stability of the AMP transparent heater. Current-time responses under constant applied voltages (1–5 V) for 10 h.


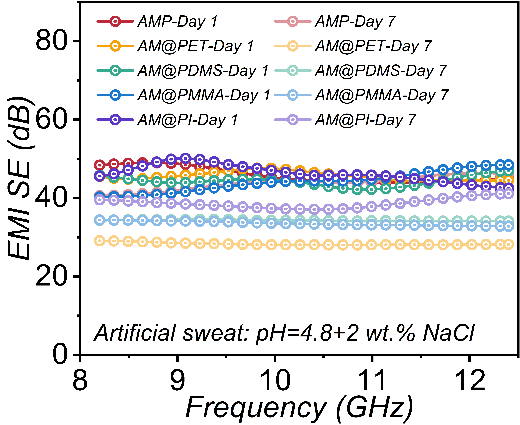


**Figure S22.** Artificial sweat corrosion resistance. EMI SE retention of AMP and reference films after immersion in artificial sweat.


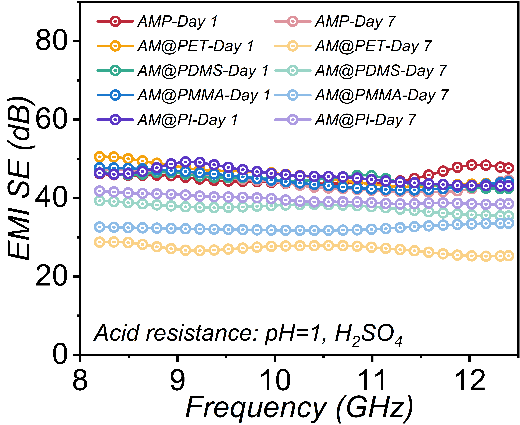


**Figure S23.** Acid corrosion resistance (pH = 1). EMI SE stability of AMP and reference films during acidic exposure.


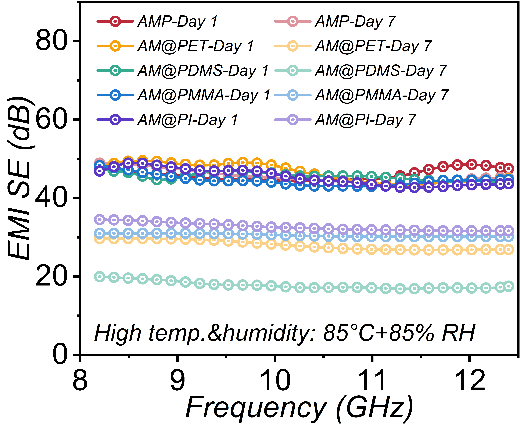


**Figure S24.** High-temperature and high-humidity aging stability (85℃/85% RH). EMI SE retention of the AMP film under high temperature and humidity.


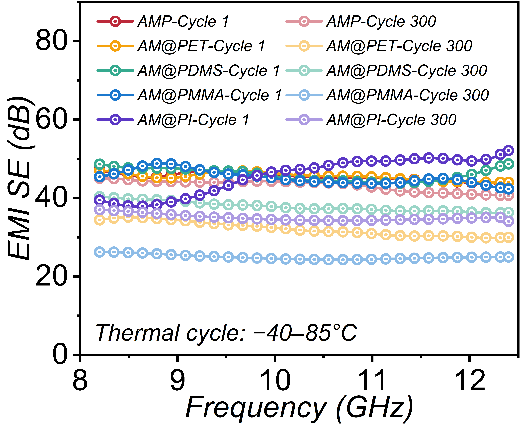


**Figure S25.** Thermal cycling durability. EMI SE retention of the AMP film under repeated thermal cycling from −40 to 85℃.


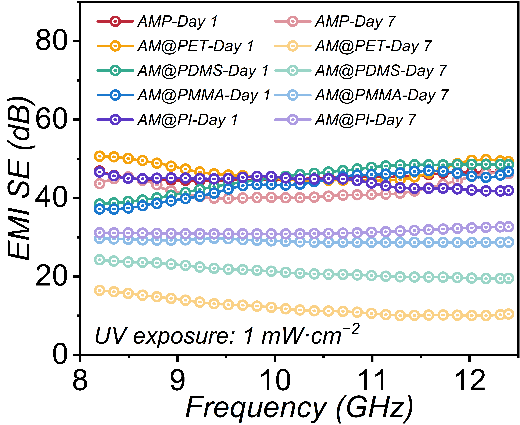


**Figure S26.** UV aging resistance. EMI SE retention under continuous UV irradiation (1 mW·cm^−2^).


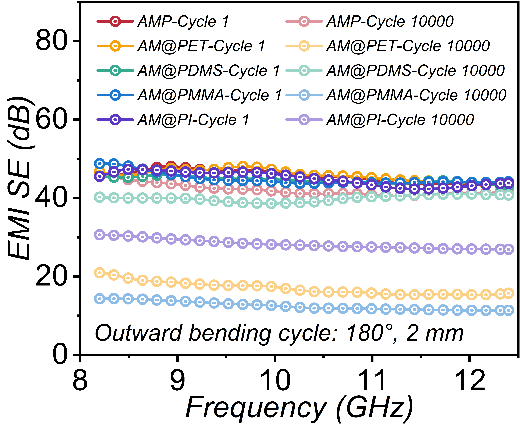


**Figure S27.** Bending fatigue resistance. EMI SE retention over repeated bending cycles (180°, 2 mm radius).


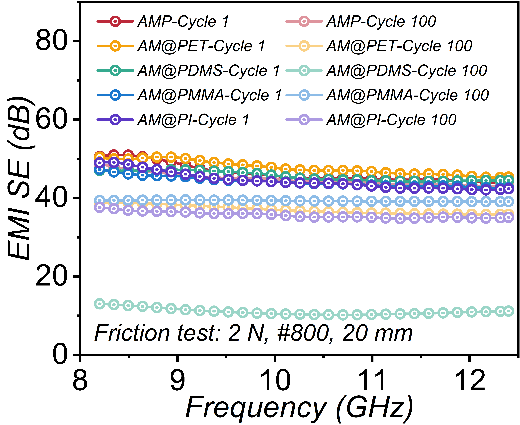


**Figure S28.** Mechanical abrasion resistance. EMI SE retention under repeated friction cycles.

**Table S1** Comparison of characteristics and performance of TCFs

| **Material** | **T_550_ (%)** | **EMI SE (dB)** | **Bandwidth (GHz)** | **Durability tests** | **Toughness (MJ·m^−3^)** | **Refs.** |
| --- | --- | --- | --- | --- | --- | --- |
| ITO | 81.5 | 18 | 12.4–18 | / | / | [51] |
| ITO/AgNWs | 71 | 37 | 5.8–12.4 | 1 | 25 | [52] |
| ITO/Ag | 87.5 | 36.5 | 8.2–26.5 | 3 | / | [53] |
| AgNWs | 72.9 | 35.2 | 8.2–26.5 | 1 | / | [54] |
| AgNWs/PI | 45 | 46 | 5.8–18 | 3 | 2.1 | [55] |
| AgNWs/PI | 83.1 | 35 | 2.2–6 | 1 | / | [56] |
| AgNWs/CA | 81 | 31.3 | 8.2–12.4 | 2 | / | [57] |
| AgNWs/PEDOT | 91 | 23 | 8.2–12.4 | / | / | [58] |
| AgNWs/TCP | 76.1 | 25.5 | 8.2–12.4 | 1 | 2.5 | [59] |
| AgNWs/Fe_3_O_4_ | 75 | 26.2 | 8.2–12.4 | 1 | / | [60] |
| AgNWs/rGO | 91.1 | 35.5 | 8.2–12.4 | 2 | / | [61] |
| AgNWs/rGO | 81.9 | 33.6 | 8.2–12.4 | 2 | / | [62] |
| AgNWs/Graphene | 16 | 26 | 12.4–18 | 1 | / | [63] |
| MXene | 42.5 | 13.7 | 8.2–12.4 | / | / | [64] |
| MXene | 43.1 | 10.9 | 8.2–12.4 | / | 70 | [65] |
| MXene/BC | 46.2 | 20.2 | 8.2–12.4 | 1 | 31.1 | [66] |
| MXene/Ni | 64 | 45 | 8.2–12.4 | 1 | / | [67] |
| MXene/SiO_2_ | 33.4 | 20.1 | 8.2–12.4 | 2 | / | [68] |
| CNTs/Ni/Pd | 71.4 | 21.4 | 8–13 | 1 | / | [69] |
| MWCNTs/MXene | 33 | 2.8 | 8.2–12.4 | 1 | 0.05 | [70] |
| SWCNTs/MXene | 41 | 3.3 | 8.2–12.4 | 1 | / |  |
| **AMP film** | **84.9** | **33.4** | **4–40**  **100–2000** | **7** | **67.2** | **This work** |

**Table S2** Comparison of transmittance and sheet resistance of TCFs

| **Material** | **T_550_ (%)** | **Rs (Ω·sq^−1^)** | **Refs.** |
| --- | --- | --- | --- |
| ITO | 81.5 | 16.5 | [51] |
| AgNWs/TCP | 53.1 | 6.4 | [59] |
|  | 76.1 | 46 |  |
| AgNWs/Fe_3_O_4_ | 75 | 16.4 | [60] |
| AgNWs/Graphene | 16 | 78.4 | [63] |
| AgNWs/PC | 52.3 | 18.3 | [71] |
| AgNWs/SiO_2_ | 31.7 | 148.5 | [72] |
| AgNWs/CNTs | 77.7 | 28.2 | [73] |
|  | 83.4 | 62.3 | [74] |
| AgNWs/rGO | 72 | 27 | [75] |
|  | 70.5 | 63 | [76] |
| MXene | 42.5 | 150 | [64] |
|  | 43.1 | 119.1 | [65] |
| MXene/SiO_2_ | 33.4 | 35.1 | [68] |
| MXene/MWCNTs | 33 | 463 | [70] |
| MXene/SWCNTs | 41 | 503 |  |
| CNTs | 88 | 100 | [77] |
|  | 71 | 59 | [78] |
|  | 80 | 100 | [79] |
| CNTs/Ni/Pd | 71.4 | 87.3 | [69] |
| CNTs/Graphene | 86 | 69 | [80] |
|  | 85.6 | 170 | [81] |
|  | 90 | 735 | [82] |
|  | 85 | 220 | [83] |
|  | 76.7 | 136 | [84] |
|  | 86.5 | 1600 | [85] |
| **AMP film** | **90.7** | **42.9** | **This work** |
|  | **89.2** | **25.0** |  |
|  | **84.8** | **15.3** |  |
|  | **78.1** | **9.5** |  |
|  | **68.0** | **4.5** |  |
|  | **50.9** | **2.2** |  |

**Table S3** Comparison of transmittance and EMI shielding effectiveness of TCFs

| **Material** | **T_550_ (%)** | **EMI SE (dB)** | **Refs.** |
| --- | --- | --- | --- |
| Cu | 82 | 24 | [51] |
| AgNWs | 92 | 17.7 | [60] |
| Ag | 88.2 | 20.75 | [86] |
| AgNWs/CA | 92 | 20.7 | [57] |
| AgNWs/TCP | 53.1 | 39.1 | [59] |
| AgNWs/PET | 81 | 25 | [87] |
| AgNWs/Fe_3_O_4_ | 75 | 26.2 | [60] |
| Ni/Pd/CNTs | 71.4 | 21.4 | [69] |
| AgNWs/GO | 85 | 24 | [88] |
| MXene | 45 | 10 | [64] |
| MXene/PVA | 52.3 | 32 | [71] |
| MXene/MWCNTs | 33 | 2.8 | [70] |
| MXene/SWCNTs | 41 | 3.3 |  |
| Graphene | 97 | 2.27 | [89] |
|  | 71 | 12.9 | [90] |
| Graphene/PET | 80.5 | 19.14 | [91] |
| Graphene/PEI | 62 | 6.4 | [92] |
| Graphene/AgNWs | 78.4 | 26 | [63] |
| Graphene/Al | 94 | 20.86 | [93] |
| **AMP film** | **90.7** | **22.8** | **This work** |
|  | **89.2** | **29.2** |  |
|  | **84.8** | **33.4** |  |
|  | **78.1** | **37.1** |  |
|  | **68.0** | **45.8** |  |
|  | **50.9** | **54.1** |  |

**Table S4** Comparison of bandwidth and EMI shielding effectiveness of TCFs

| **Material** | **Bandwidth (GHz)** | **EMI SE (dB)** | **Refs.** |
| --- | --- | --- | --- |
| Cu | 6 | 24 | [51] |
| AgNWs | 4 | 17.7 | [90] |
| Ag | 10 | 26 | [86] |
| AgNWs/CA | 4 | 20.7 | [57] |
| AgNWs/TCP | 4.2 | 39.1 | [59] |
| AgNWs/PET | 4 | 25 | [87] |
| AgNWs/Fe_3_O_4_ | 4 | 26.2 | [60] |
| Ni/Pd/CNTs | 7 | 21.4 | [69] |
| AgNWs/GO | 2.5 | 24 | [88] |
| MXene | 4.2 | 10 | [64] |
| MXene/PVA | 4.2 | 32 | [71] |
| MXene/MWCNTs | 5.6 | 2.8 | [70] |
| MXene/SWCNTs | 4.2 | 3.3 |  |
| Graphene | 4.8 | 2.27 | [89] |
|  | 5.8 | 12.9 | [90] |
| Graphene/PET | 8.5 | 19.14 | [91] |
| Graphene/PEI | 8 | 6.4 | [92] |
| Graphene/AgNWs | 5.6 | 26 | [63] |
| Graphene/Al | 6 | 20.86 | [93] |
| Ni/PVDF | 8.5 | 35.4 | [94] |
| Cu/Ag/ITO | 31.8 | 26 | [95] |
| AgNWs | 2500 | 8 | [96] |
| MXene | 4.2 | 48 | [97] |
| Graphene/PMMA | 1800 | 15 | [98] |
| CNTs | 2300 | 8 | [99] |
| **AMP film** | **1936** | **33.4** | **This work** |

**Reference**

[51] Y. Han, H. Zhong, N. Liu, Y. Liu, J. Lin, P. Jin, "In Situ Surface Oxidized Copper Mesh Electrodes for High-Performance Transparent Electrical Heating and Electromagnetic Interference Shielding," *Adv. Electron. Mater.* **4**, (2018): 1800156, https://doi.org/10.1002/aelm.201800156.

[52] Q. Chen, L. Huang, X. Wang, Y. Yuan, "Transparent and Flexible Composite Films with Excellent Electromagnetic Interference Shielding and Thermal Insulating Performance," *ACS Appl. Mater. Interfaces* **15**, (2023): 24901–24912, https://doi.org/10.1021/acsami.3c03140.

[53] B. Feng, T. Yang, S. Zhu, Y. Yue, B. Zhou, Z. Yao, C. Liu, J. Han, "Flexible, Highly Transparent, and Conductive ITO/Ag/ITO Film with Double Symmetric Structure for Electrical Heater and Broadband Electromagnetic Interference Shielding," *Chem. Eng. J.* **516**, (2025): 164233, https://doi.org/10.1016/j.cej.2025.164233.

[54] W. Zhao, J. Dong, Z. Li, B. Zhou, C. Liu, Y. Feng, "Centrifugal Inertia-Induced Directional Alignment of AgNW Network for Preparing Transparent Electromagnetic Interference Shielding Films with Joule Heating Ability," *Adv. Sci.* **11**, (2024): 2406758, https://doi.org/10.1002/advs.202406758.

[55] Z. Huang, Y. Xin, J. Shen, L. Shen, J. Liu, X. Zeng, H. Ling, G. Tu, H. Yang, D. He, B. Hu, "Transparent and Lightweight Electromagnetic Shielding Film with Resistance to Harsh Conditions Through Interpenetrating Encapsulation of Silver Nanowires Network," *Chem. Eng. J.* **505**, (2025): 159382, https://doi.org/10.1016/j.cej.2025.159382.

[56] J. Zhang, V. Selamneni, B. P. Yalagala, B. King, J. Wang, L. Khurelbaatar, C. G. Núñez, M. Wagih, M. Amjadi, H. Heidari, "Laser-Engineered Interfacial Dielectrophoresis-Aligned Nanowire Networks for Transparent Electromagnetic Interference-Shielding Films," *ACS Nano* **19**, (2025): 42760–42771, https://doi.org/10.1021/acsnano.5c13772.

[57] L. Jia, D. Yan, X. Liu, R. Ma, H. Wu, Z. Li, "Highly Efficient and Reliable Transparent Electromagnetic Interference Shielding Film," *ACS Appl. Mater. Interfaces* **10**, (2018): 11941–11949, https://doi.org/10.1021/acsami.8b00492.

[58] E. Hosseini, N. Sabet, M. Arjmand, U. Sundararaj, H. Hassanzadeh, M. H. Zarifi, K. Karan, "Multilayer Polymeric Nanocomposite Thin Film Heater and Electromagnetic Interference Shield," *Chem. Eng. J.* **435**, (2022): 134598, https://doi.org/10.1016/j.cej.2022.134598.

[59] M. Zhu, X. Yan, X. Li, L. Dai, J. Guo, Y. Lei, Y. Xu, H. Xu, "Flexible, Transparent, and Hazy Composite Cellulosic Film with Interconnected Silver Nanowire Networks for EMI Shielding and Joule Heating," *ACS Appl. Mater. Interfaces* **14**, (2022): 45697–45706, https://doi.org/10.1021/acsami.2c13035.

[60] Z. Wang, B. Jiao, Y. Qing, H. Nan, L. Huang, W. Wei, Y. Peng, F. Yuan, H. Dong, X. Hou, Z. Wu, "Flexible and Transparent Ferroferric Oxide-Modified Silver Nanowire Film for Efficient Electromagnetic Interference Shielding," *ACS Appl. Mater. Interfaces* **12**, (2020): 2826–2834, https://doi.org/10.1021/acsami.9b17513.

[61] Y. Yang, S. Chen, W. Li, P. Li, J. Ma, B. Li, X. Zhao, Z. Ju, H. Chang, L. Xiao, H. Xu, Y. Liu, "Reduced Graphene Oxide Conformally Wrapped Silver Nanowire Networks for Flexible Transparent Heating and Electromagnetic Interference Shielding," *ACS Nano* **14**, (2020): 8754–8765, https://doi.org/10.1021/acsnano.0c03337.

[62] G. Wang, Y. Zhao, F. Yang, Y. Zhang, M. Zhou, G. Ji, "Multifunctional Integrated Transparent Film for Efficient Electromagnetic Protection," *Nano-Micro Lett.* **14**, (2022): 65, https://doi.org/10.1007/s40820-022-00810-y.

[63] N. Zhang, Z. Wang, R. Song, Q. Wang, H. Chen, B. Zhang, H. Lv, Z. Wu, D. He, "Flexible and Transparent Graphene/Silver-Nanowires Composite Film for High Electromagnetic Interference Shielding Effectiveness," *Sci. Bull.* **64**, (2019): 540–546, https://doi.org/10.1016/j.scib.2019.03.028.

[64] T. Yun, H. Kim, A. Iqbal, Y. S. Cho, G. S. Lee, M. Kim, S. J. Kim, D. Kim, Y. Gogotsi, S. O. Kim, C. M. Koo, "Electromagnetic Shielding of Monolayer MXene Assemblies," *Adv. Mater.* **32**, (2020): 1906769, https://doi.org/10.1002/adma.201906769.

[65] Q. Li, Y. Sun, B. Zhou, G. Han, Y. Feng, C. Liu, C. Shen, "Flexible, Stretchable, and Transparent MXene Nanosheet/Thermoplastic Polyurethane Films for Multifunctional Heating and Electromagnetic Interference Shielding," *ACS Appl. Nano Mater.* **6**, (2023): 3395–3404, https://doi.org/10.1021/acsanm.2c05169.

[66] C. Ma, W. Cao, W. Zhang, M. Ma, W. Sun, J. Zhang, F. Chen, "Wearable, Ultrathin and Transparent Bacterial Celluloses/MXene Film with Janus Structure and Excellent Mechanical Property for Electromagnetic Interference Shielding," *Chem. Eng. J.* **403**, (2021): 126438, https://doi.org/10.1016/j.cej.2020.126438.

[67] K. Kim, J. Kim, F. B. Iniguez, H. Kim, M. W. Lee, J. Noh, S. An, "Percolative Network-Based Flexible Transparent Conductive MXene-Nickel Microfiber Film for Electromagnetic Interference Shielding," *ACS Nano* **19**, (2025): 31753–31767, https://doi.org/10.1021/acsnano.5c09918.

[68] B. Zhou, Z. Li, Y. Li, X. Liu, J. Ma, Y. Feng, D. Zhang, C. He, C. Liu, C. Shen, "Flexible Hydrophobic 2D Ti_3_C_2_T_x_-Based Transparent Conductive Film with Multifunctional Self-Cleaning, Electromagnetic Interference Shielding and Joule Heating Capacities," *Compos. Sci. Technol.* **201**, (2021): 108531, https://doi.org/10.1016/j.compscitech.2020.108531.

[69] J. Park, H. Rho, A. Cha, H. Bae, S. H. Lee, S. Ryu, T. Jeong, J. Ha, "Transparent Carbon Nanotube Web Structures with Ni-Pd Nanoparticles for Electromagnetic Interference (EMI) Shielding of Advanced Display Devices," *Appl. Surf. Sci.* **516**, (2020): 145745, https://doi.org/10.1016/j.apsusc.2020.145745.

[70] G. Weng, J. Li, M. Alhabeb, C. Karpovich, H. Wang, J. Lipton, K. Maleski, J. Kong, E. Shaulsky, M. Elimelech, Y. Gogotsi, A. D. Taylor, "Layer-by-Layer Assembly of Cross-Functional Semi-transparent MXene-Carbon Nanotubes Composite Films for Next-Generation Electromagnetic Interference Shielding," *Adv. Funct. Mater.* **28**, (2018): 1803360, https://doi.org/10.1002/adfm.201803360.

[71] B. Zhou, M. Su, D. Yang, G. Han, Y. Feng, B. Wang, J. Ma, J. Ma, C. Liu, C. Shen, "Flexible MXene/Silver Nanowire-Based Transparent Conductive Film with Electromagnetic Interference Shielding and Electro-Photo-Thermal Performance," *ACS Appl. Mater. Interfaces* **12**, (2020): 40859–40869, https://doi.org/10.1021/acsami.0c09020.

[72] Z. Li, W. Che, Y. Jiang, Y. Liu, X. Fang, Y. Peng, "Strong, Hydrophobic, and Transparent Wood Film Decorated with MXene/Silver Nanowire for Electromagnetic Interference Shielding and Electrothermal Conversion," *Colloids Surf. A* **676**, (2023): 132211, https://doi.org/10.1016/j.colsurfa.2023.132211.

[73] J. Goak, T. Kim, D. Kim, K. Chang, C. Lee, N. Lee, "Stable Heating Performance of Carbon Nanotube/Silver Nanowire Transparent Heaters," *Appl. Surf. Sci.* **510**, (2020): 145445, https://doi.org/10.1016/j.apsusc.2020.145445.

[74] S. Lee, J. Kim, J. Park, Y. Porte, J. Kim, J. Park, S. Kim, J. Myoung, "SWCNT-Ag Nanowire Composite for Transparent Stretchable Film Heater with Enhanced Electrical Stability," *J. Mater. Sci.* **53**, (2018): 12284–12294, https://doi.org/10.1007/s10853-018-2526-7.

[75] P. Meenakshi, R. Karthick, M. Selvaraj, S. Ramu, "Investigations on Reduced Graphene Oxide Film Embedded with Silver Nanowire as a Transparent Conducting Electrode," *Sol. Energy Mater. Sol. Cells* **128**, (2014): 264–269, https://doi.org/10.1016/j.solmat.2014.05.013.

[76] J. Miao, H. Liu, W. Li, X. Zhang, "Mussel-Inspired Polydopamine-Functionalized Graphene as a Conductive Adhesion Promoter and Protective Layer for Silver Nanowire Transparent Electrodes," *Langmuir* **32**, (2016): 5365–5372, https://doi.org/10.1021/acs.langmuir.6b00796.

[77] I. Jeon, J. Yoon, U. Kim, C. Lee, R. Xiang, A. Shawky, J. Xi, J. Byeon, H. M. Lee, M. Choi, S. Maruyama, Y. Matsuo, "High-Performance Solution-Processed Double-Walled Carbon Nanotube Transparent Electrode for Perovskite Solar Cells," *Adv. Energy Mater.* **9**, (2019): 1901204, https://doi.org/10.1002/aenm.201901204.

[78] J. Jo, J. Jung, J. Lee, W. Jo, "Fabrication of Highly Conductive and Transparent Thin Films from Single-Walled Carbon Nanotubes Using a New Non-ionic Surfactant via Spin Coating," *ACS Nano* **4**, (2010): 5382–5388, https://doi.org/10.1021/nn1009837.

[79] Y. Wang, S. Tong, X. Xu, B. Özyilmaz, K. Loh, "Interface Engineering of Layer-by-Layer Stacked Graphene Anodes for High-Performance Organic Solar Cells," *Adv. Mater.* **23**, (2011): 1514–1518, https://doi.org/10.1002/adma.201003673.

[80] Y. Yue, D. Zhang, P. Wang, X. Xia, X. Wu, Y. Zhang, J. Mei, S. Li, M. Li, Y. Wang, X. Zhang, X. Wei, H. Liu, W. Zhou, "Large-Area Flexible Carbon Nanofilms with Synergistically Enhanced Transmittance and Conductivity Prepared by Reorganizing Single-Walled Carbon Nanotube Networks," *Adv. Mater.* **36**, (2024): 2313971, https://doi.org/10.1002/adma.202313971.

[81] E. Shi, H. Li, W. Xu, S. Wu, J. Wei, Y. Fang, A. Cao, "Improvement of Graphene-Si Solar Cells by Embroidering Graphene with a Carbon Nanotube Spider-Web," *Nano Energy* **17**, (2015): 216–223, https://doi.org/10.1016/j.nanoen.2015.08.018.

[82] C. Li, Z. Li, H. Zhu, K. Wang, J. Wei, X. Li, P. Sun, H. Zhang, D. Wu, "Graphene Nano-'patches' on a Carbon Nanotube Network for Highly Transparent/Conductive Thin Film Applications," *J. Phys. Chem. C* **114**, (2010): 14008–14012, https://doi.org/10.1021/jp1041487.

[83] I. N. Kholmanov, C. W. Magnuson, R. Piner, J. Kim, A. E. Aliev, C. Tan, T. Kim, A. A. Zakhidov, G. Sberveglieri, R. H. Baughman, R. S. Ruoff, "Optical, Electrical, and Electromechanical Properties of Hybrid Graphene/Carbon Nanotube Films," *Adv. Mater.* **27**, (2015): 3053–3059, https://doi.org/10.1002/adma.201500785.

[84] S. Yadav, V. Kumar, S. Arora, S. Singh, D. Bhatnagar, I. Kaur, "Fabrication of Ultrathin, Free-Standing, Transparent and Conductive Graphene/Multiwalled Carbon Nanotube Film with Superior Optoelectronic Properties," *Thin Solid Films* **595**, (2015): 193–199, https://doi.org/10.1016/j.tsf.2015.11.002.

[85] C. Feng, K. Liu, J. Wu, L. Liu, J. Cheng, Y. Zhang, Y. Sun, Q. Li, S. Fan, K. Jiang, "Flexible, Stretchable, Transparent Conducting Films Made from Superaligned Carbon Nanotubes," *Adv. Funct. Mater.* **20**, (2010): 885–891, https://doi.org/10.1002/adfm.200901960.

[86] S. K. Vishwanath, D. Kim, J. Kim, "Electromagnetic Interference Shielding Effectiveness of Invisible Metal-Mesh Prepared by Electrohydrodynamic Jet Printing," *Jpn. J. Appl. Phys.* **53**, (2014): 05HB11, https://doi.org/10.7567/JJAP.53.05HB11.

[87] M. Hu, J. Gao, Y. Dong, K. Li, G. Shan, S. Yang, R. K. Li, "Flexible Transparent PES/Silver Nanowires/PET Sandwich-Structured Film for High-Efficiency Electromagnetic Interference Shielding," *Langmuir* **28**, (2012): 7101–7106, https://doi.org/10.1021/la300720y.

[88] D. Kim, J. Choi, D. Choi, S. Kim, "Highly Bendable and Durable Transparent Electromagnetic Interference Shielding Film Prepared by Wet Sintering of Silver Nanowires," *ACS Appl. Mater. Interfaces* **10**, (2018): 29730–29740, https://doi.org/10.1021/acsami.8b07054.

[89] S. Hong, K. Kim, T. Kim, J. Kim, S. Park, J. Kim, B. Cho, "Electromagnetic Interference Shielding Effectiveness of Monolayer Graphene," *Nanotechnology* **23**, (2012): 455704, https://doi.org/10.1088/0957-4484/23/45/455704.

[90] J. Han, X. Wang, Y. Qiu, J. Zhu, P. Hu, "Infrared-Transparent Films Based on Conductive Graphene Network Fabrics for Electromagnetic Shielding," *Carbon* **87**, (2015): 206–214, https://doi.org/10.1016/j.carbon.2015.01.057.

[91] Z. Lu, L. Ma, J. Tan, H. Wang, X. Ding, "Transparent Multi-Layer Graphene/Polyethylene Terephthalate Structures with Excellent Microwave Absorption and Electromagnetic Interference Shielding Performance," *Nanoscale* **8**, (2016): 16684–16693, https://doi.org/10.1039/c6nr02619b.

[92] S. Kim, J. Oh, M. Kim, W. Jang, M. Wang, Y. Kim, H. Seo, Y. Kim, J. Lee, Y. Lee, J. Nam, "Electromagnetic Interference (EMI) Transparent Shielding of Reduced Graphene Oxide (RGO) Interleaved Structure Fabricated by Electrophoretic Deposition," *ACS Appl. Mater. Interfaces* **6**, (2014): 17647–17653, https://doi.org/10.1021/am503893v.

[93] L. Ma, Z. Lu, J. Tan, J. Liu, X. Ding, N. Black, T. Li, J. Gallop, L. Hao, "Transparent Conducting Graphene Hybrid Films To Improve Electromagnetic Interference (EMI) Shielding Performance of Graphene," *ACS Appl. Mater. Interfaces* **9**, (2017): 34221–34229, https://doi.org/10.1021/acsami.7b09372.

[94] B. Zhao, C. Park, "Tunable Electromagnetic Shielding Properties of Conductive Poly(vinylidene Fluoride)/Ni Chain Composite Films with Negative Permittivity," *J. Mater. Chem. C* **5**, (2017): 6954–6961, https://doi.org/10.1039/c7tc01865g.

[95] J. Jung, H. Lee, I. Ha, H. Cho, K. Kim, J. Kwon, P. Won, S. Hong, S. Ko, "Highly Stretchable and Transparent Electromagnetic Interference Shielding Film Based on Silver Nanowire Percolation Network for Wearable Electronics Applications," *ACS Appl. Mater. Interfaces* **9**, (2017): 44609–44616, https://doi.org/10.1021/acsami.7b14626.

[96] N. V. Hoof, M. Parente, A. Baldi, J. G. Rivas, "Terahertz Time-Domain Spectroscopy and Near-Field Microscopy of Transparent Silver Nanowire Networks," *Adv. Opt. Mater.* **8**, (2020): 1900790, https://doi.org/10.1002/adom.201900790.

[97] J. Wang, X. Ma, J. Zhou, F. Du, C. Teng, "Bioinspired, High-Strength, and Flexible MXene/Aramid Fiber for Electromagnetic Interference Shielding Papers with Joule Heating Performance," *ACS Nano* **16**, (2022): 6700–6711, https://doi.org/10.1021/acsnano.2c01323.

[98] C. Pavlou, M. G. P. Carbone, A. C. Manikas, G. Trakakis, C. Koral, G. Papari, A. Andreone, C. Galiotis, "Effective EMI Shielding Behaviour of Thin Graphene/PMMA Nanolaminates in the THz Range," *Nat. Commun.* **12**, (2021): 4655, https://doi.org/10.1038/s41467-021-24970-4.

[99] M. Seo, J. Yim, Y. Ahn, F. Rotermund, D. Kim, S. Lee, H. Lim, "Terahertz Electromagnetic Interference Shielding Using Single-Walled Carbon Nanotube Flexible Films," *Appl. Phys. Lett.* **93**, (2008): 231905, https://doi.org/10.1063/1.3046126.
